# H_2_O_2_/DEM-Promoted *Maft* Promoter Demethylation Drives Nrf2/ARE Activation in Zebrafish

**DOI:** 10.3390/life12091436

**Published:** 2022-09-15

**Authors:** Ce Chen, Mingyue He, Xueting Li, Lidong Yu, Yi Liu, Yan Yang, Li Li, Jianbo Jia, Bingsheng Li

**Affiliations:** 1 School of Life Science and Technology, Harbin Institute of Technology, Harbin 150080, China; 2 School of Physics, Harbin Institute of Technology, Harbin 150080, China; 3 School of Biotechnology and Health Sciences, Wuyi University, Jiangmen 529020, China; 4 Key Laboratory of UV Light Emitting Materials and Technology of Ministry of Education, Northeast Normal University, Changchun 130024, China

**Keywords:** zebrafish, Nrf2/ARE signaling pathway, oxidative stress, methylation regulation

## Abstract

The Nrf2/ARE signaling pathway is a cell survival response pathway in response to environmental stresses. The Nrf2/ARE signaling pathway can be activated by stimulating cysteine residues at different positions in the Keap1. However, the epigenetic mechanisms of the Nrf2/ARE pathway under different stimuli are still poorly understood. In this study, we found that both hydrogen peroxide (H_2_O_2_) and Diethyl Maleate (DEM) activated the Nrf2/ARE signaling pathway at 120 hpf in zebrafish. H_2_O_2_ regulated the demethylation of the *maft* promoter by inhibiting the expression of methyltransferase. This promotes the mRNA expression of the Nrf2 binding factor *maft*, thereby promoting the downstream antioxidant genes. The methylation of the Nrf2/ARE signaling pathway was not significantly regulated by DEM. However, under oxidative stress, the methyltransferase inhibitors (decitabine and azacitidine) demethylated the promoter region of *maft*. It activated the expression of the *maft*, further improving the Nrf2/ARE signal pathway. At last, antioxidant target genes were activated. It was shown that H_2_O_2_ and DEM cooperated with methyltransferase inhibitors, providing an important reference for the treatment of oxidative stress-related diseases and breaking new ground for the study of the mechanism of methyltransferase inhibitors in the process of tumor chemotherapy.

## 1. Introduction

Maintaining redox levels is an important factor to ensure cell homeostasis. However, this complex balance is constantly disturbed by various environmental oxidants and electrophiles, such as pollutants, food additives, ultraviolet light, and ionizing radiation [1]. In addition, reactive oxygen species (ROS), including superoxide anions (O^2−^), hydroxyl radicals (OH-), and H_2_O_2_, can severely disrupt cellular homeostasis [2]. Chronic exposure to electrophilic and oxidative stress environments can lead to a variety of diseases, such as cancer, cardiovascular diseases, and neurodegenerative disorders [3,4]. ROS, such as OH-, can cause severe DNA damage and mutations, which can also lead to overall DNA hypomethylation [5]. The Nrf2/ARE signaling pathway is an important defense system for antioxidant and electrophilic stress in vivo. The transcription factor nuclear E2-factor-related factor 2 (Nrf2) is key to the antioxidant response. Under normal conditions, Nrf2 is ubiquitinated at the cell membrane by Kelch-like ECH-associated protein 1 (Keap1) in the cytoplasm [6]. However, when the organism is exposed to electrophiles and oxidative stress, Nrf2 dissociates from Keap1 and rapidly translocates into the nucleus to form a heterodimer with sMaf protein, while binding with ARE to activate the transcription of antioxidant genes [7].

The exploration of the DNA methylation of target genes downstream of the Nrf2 signaling pathway has attracted the attention of many researchers. DNA methylation can regulate gene transcription. Generally, DNA methylation is negatively correlated with gene expression and plays an important role in early embryonic growth and development and various diseases [8]. The regulation of DNA methylation mainly occurs in the promoter region of genes, which means that the covalent modification of methylcytosine and histone is added to the specific promoter region to change the structure of DNA and chromatin and finally achieve the process of regulating gene transcription [9].

The promoter region of many genes is usually enriched in some nucleotide “CG”. In addition, the content of CG is greater than or equal to 50%, and the sequence length is more than 200 bp, which is commonly referred to as a “CpG island”. About 60% of human genes contain CpG islands in the promoter region [10]. The methylation of CpG islands in the gene promoter region can change the chromatin structure or inhibit its binding with transcription factors, resulting in the cessation of gene transcription or the downregulation of the transcription level [11]. Most CpG islands are unmethylated in the human genome and are located in the transcriptional regulatory region, which is closely related to the coding genes of the genome under normal circumstances. Therefore, it is very important to study the methylation of CpG islands in the transcriptional region.

**Scheme 1:**
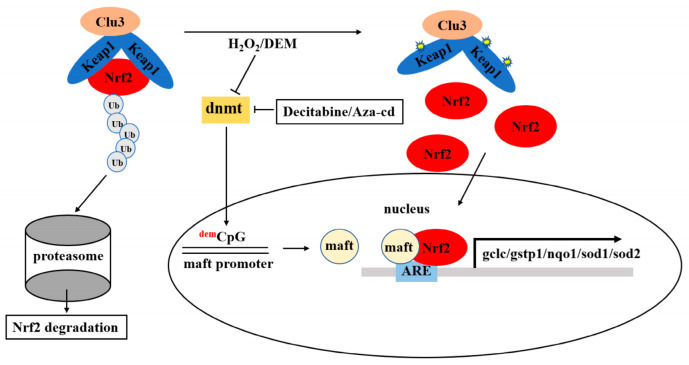
Mechanism of H_2_O_2_/DEM-induced activation of Nrf2/ARE signaling pathway.

Almost all cancers have abnormal DNA methylation [12,13]. DNA methylation studies of zebrafish are rapidly emerging in the field of epigenetics because of the economic and ethical considerations involved in epigenetic studies using rodent models [14]. In mammals, DNA methylation is modeled under embryonic development and certain physiological and pathological conditions and is catalyzed by DNA methyltransferase [15]. Zebrafish have Keap1 and Nrf2, which are homologous to mammals [16]. Using zebrafish as the research object, the regulation of the Nrf2/ARE signaling pathway and the mechanism leading to related diseases can be further studied [17]. At present, how this signaling pathway is regulated by DNA methylation in zebrafish models has not been studied in detail. Therefore, zebrafish is used as a model organism in this study to explore the mechanism of the activation of the Nrf2/ARE signaling pathway induced by H_2_O_2_/DEM, as shown in Scheme 1. It was found that the expression of the small Maf family gene *maft* was regulated by the methylation of its gene promoter region and the activated downstream target gene expression of the Nrf2/ARE signaling pathway. At the same time, methyltransferase inhibitors were used to demethylate the promoter region of *maft*, which increased the transcriptional expression of *maft* and promoted the transcription of the downstream target genes of the Nrf2/ARE signaling pathway.

## 2. Materials and Methods

### 2.1. Materials and Instruments

Zebrafish is a wild-type strain AB, which was purchased from the Heilongjiang Institute of Fisheries, Chinese Academy of Fisheries Sciences. H_2_O_2_, RNaseA, protease K, ethylenediaminetetraacetic acid (EDTA), and sodium dodecyl sulfate (SDS) were purchased from Fuyu Fine Chemical Co., Ltd.(Tianjin, China). Trizol reagent was purchased from Dalian Bao Biological Co., Ltd. (Dalian, China). X-Gal, DEPC, Tris-HCl was purchased from Biotopped (Chicago, IL, USA). DEM was purchased from Wako Pure Chemical Industries, Ltd. (Osaka, Japan). Agar, IPTG, DMSO, and agarose were purchased from Aladdin Reagent Co., Ltd. (Shanghai, China). Trizol, a PrimeScript^TM^ RT reagent Kit with gDNA Eraser, Premix TaqTM, and SYBR^®^ Premix Ex TaqTM II (Tli RNaseH Plus) were bought from Harbin Baoste Biotechnology Co., Ltd. (Takara, China). An Animal Genomic DNA Quick Extraction Kit was obtained from Beyotime Biotechnology Co., Ltd. (Shanghai, China). An EZ DNA Methylation-Direct KitTM (D5020) kit was purchased from ZYMO RESEARCH Co., Ltd. (Beijing, China). A DNA Gel Extraction Kit was from AxyPrep. Methyltransferase inhibitor: decitabine was purchased from Solarbio Co., Ltd. (Beijing, China), and azacitidine was purchased from Shanghai Ruiyong Biotechnology Co., Ltd. (Shanghai, China). PCR primers were synthesized by BGI and Jilin Kumei Biotechnology Co., Ltd. (Changchun, China), as shown in Table S1. Gene sequencing was performed by Jilin Kumei Biotechnology Co., Ltd. (Changchun, China).

The automatic light-controlled circulating water system for zebrafish feeding was purchased from Taiwan Chuanfu Science and Technology Development Co., Ltd. (Taiwan, China); the SPX-250BIII biochemical incubator of the Tester was used for embryo culture. Sterilization operation used GI54DWS type autoclave. qRT-PCR was performed using Applied Biosystems ABI 7500. DNA and RNA purity analysis used Thermo Fisher NanoDrop 2000. A Gel imaging system Tanon 1600 was purchased from Tanon Science and Technology Co., Ltd. (Shanghai, China). Other conventional equipment included a metal bath, water bath, centrifuge, electronic balance, electrophoresis apparatus, etc.

### 2.2. H_2_O_2_ and DEM Exposure of Zebrafish

Male: female = 2:1, mating and spawning. The next day, fertilized eggs were selected and placed in six-well plates, 40 eggs per well, and incubated in a thermostat incubator at 28 °C. Randomly take 20 embryos or larvae in 8 hpf, 12 hpf, 24 hpf, 48 hpf, 120 hpf, and 168 hpf at six time points from the control group; these are used as samples for testing the expression of the Nrf2/ARE signal pathway-related genes in different developmental stages. In addition, take 20 larvae samples at 120 hpf from three groups to detect the expression of the Nrf2/ARE signal pathway-related genes and antioxidant target genes.

**Scheme 2:**
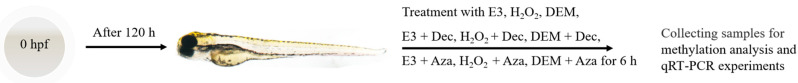
Experimental flow chart.

After 120 hpf, 1 mmol/L H_2_O_2_ and 100 µmol/L DEM (Diethyl Maleate) solution 4 mL were added for exposure experimentation, respectively. E3 water was used as a control group, cultured at 28 °C for 6 h, and samples were collected for subsequent methylation analysis and qRT-PCR experiments. Nine groups were designed, including E3 water control, 1 mmol/L H_2_O_2_, 100 µmol/L DEM (Diethyl Maleate), E3 water +3 µmol/L decitabine, 1 mmol/L H_2_O_2_ + 3 µmol/L decitabine, 100 µmol/L DEM + 3 µmol/L decitabine, E3 water +3 µmol/L azacitidine, 1 mmol/L H_2_O_2_ + 3 µmol/L azacitidine, and 100 µmol/L DEM + 3 µmol/L azacitidine for 6 h at 120 hpf, as shown in Scheme 2. Next, samples were collected for subsequent methylation analysis and qRT-PCR experiments.

### 2.3. Total RNA Extraction

Total RNA was isolated using Trizols reagent (Invitrogen) according to the following procedure. Add 800 μL of Trizol to each group of samples for homogenization, put on ice for 5 min, add 200 μL of chloroform, shake for 15 s on a vortex oscillator, keep at room temperature for 5 min, followed by centrifugation at 12,000× *g* at 4 °C for 15 min. After centrifugation, remove supernatant to a 1.5 mL centrifuge tube, add 500 uL isopropyl alcohol, and mix, keep at room temperature for 10 min, 12,000× *g*, at 4 °C for 15 min. Discard supernatant, add pre-cooled 75% ethanol (DEPC configuration), mix, and centrifuge 7500× *g* at 4 °C for 5 min. Remove the supernatant, dry naturally in a sterile environment, dissolve DEPC in water, and store at −80 °C.

### 2.4. Real-Time Quantitative PCR Assay

cDNA was obtained by the reverse transcription of the extracted zebrafish sample RNA using a PrimeScriptTM RT Reagent Kit with a gDNA Eraser Kit. Gene expression was determined by an SYBR^®^Premix Ex TaqTM II Kit (TaKaRa) that was conducted on an Applied Biosystems Prism 7500 Sequence Detection System (Applied Biosystems, Foster City, CA, USA). The primer sequences of each gene used in this analysis are shown in Table S2. PCR amplification was performed in duplicate, using the following thermal profile: initial denaturation at 95 °C for 30 s, followed by 44 cycles of 95 °C for 15 s, 60 °C for 30 s, and 72 °C for 30 s. Four parallel experiments were set up for each sample. *Ef1α* was used as a housekeeping gene. The experimental data were processed by a 2^−ΔΔCT^ method.

### 2.5. Genomic DNA Methylation Analysis

Genomic DNA extraction used Animal Genomic DNA Quick Extraction Kit for PCR Analysis (Beyotime). The DNA concentration and quality were evaluated by measuring the ratio of optical density at 260/280 nm with NanoDrop 2000. The extracted genomic DNA was methylated using EZ DNA methylation-Direct KitTM (D5020) according to the manufacturer’s protocols. The methylation-specific primer used to target the CpG islands located in the putative promoter region were shown in Table S2. These primer sets were designed using Methyl Primer Express Software v1.0 (Applied Biosystems). PCR amplification was performed using the following conditions: 95 °C 5 min, 95 °C 30 s, X °C 30 s, 72 °C 30 s, Y cycles, 72 °C 10 min (X and Y were determined by genes). PCR amplification products were purified by an AxyPrep DNA Gel Extraction Kit. The DNA methylated PCR products were recombined into the PMD-19T vector. The positive clones were selected by blue and white class screening. DNA methylation was analyzed by sequencing.

### 2.6. Statistical Analyze Software and Websites

Genetic database: http://genome.ucsc.edu/index.htmL, http://www.ncbi.nlm.nih.gov/ (accessed on 5 June 2016)

DNA sequence alignment software: DNAMAN 6.0

Methylation analysis: http://www.urogene.org/methprimer/index1.htmL, Methyl Primer Express Software v1.0, BIQ Analyzer, http://medgen.ugent.be/methBLAST/ (accessed on 3 September 2016)

qRT-PCR primer design software: NCBI primer BLAST (online) https://www.ncbi.nlm.nih.gov/tools/primer-blast/index.cgi?LINK_LOC=BlastHome (accessed on 15 July 2016)

Mapping and data analysis software: Adobe Illustrator CC 2018, GraphPad Prism 8

Statistical analyses: All data, if applicable, were expressed as mean ± SEM from at least three independent experiments by GraphPad Prism 8. Group differences were performed using a two-tailed t-test or two-way ANOVA by GraphPad Prism 8. * *p* < 0.05, ** *p* < 0.01, *** *p* < 0.001 were considered statistically significant.

## 3. Results

### 3.1. H_2_O_2_ and DEM Activate the Zebrafish Nrf2/ARE Signaling Pathway

**Figure 1:**
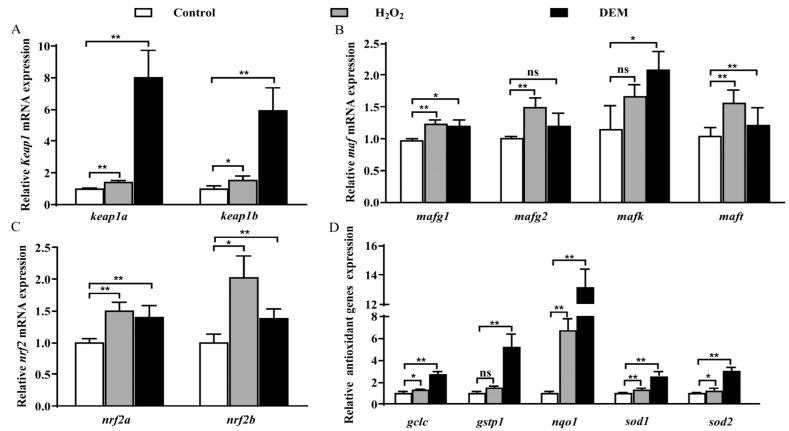
H_2_O_2_/DEM induced the activation of the Keap1/Nrf2 signaling pathway and the transcription of target genes. The changes in the relative mRNA expression of (**A**) *keap1a* and *keap1b*, (**B**) *sMaf* family genes, (**C**) *nrf2a* and *nrf2b*, (**D**) antioxidation-related genes were analyzed before and after treatment. n = 30, ** *p* < 0.01, * *p* < 0.05, ns *p* > 0.05, mean ± SEM.

The expression of genes in the Nrf2/ARE signaling pathway in 120 hpf zebrafish treated with Control (E3 water), 1 mmol/L H_2_O_2_, and 100 µmol/L DEM were detected by qRT- PCR, as shown in Figure 1A,C. Compared with the control group, the expression of *keap1a*, *keap1b*, *nrf2a*, and *nrf2b* was significantly increased under H_2_O_2_ and DEM treatment. Nrf2 cannot bind to the antioxidant response element (ARE) alone. It must form a heterodimer with Small Maf protein to initiate the transcriptional expression of downstream antioxidant target genes. Therefore, we explored the expression of the Small Maf gene family under different conditions. We found that, compared with the control group, the expression of *mafg1*, *mafg2*, and *maft* was significantly increased under H_2_O_2_ treatment, while *mafk* did not change significantly. Under DEM treatment, the expression of *mafg1*, *mafk*, and *maft* increased significantly, and there was no significant difference in *mafg2*, as shown in Figure 1B. Among them, the expression of both *maft* and *mafg1* increased significantly. Meanwhile, the expression of *gclc*, *nqo1*, *sod1*, and *sod2* significantly increased under H_2_O_2_ treatment, while the expression of *gstp1* had no significant change. Under DEM treatment, the expression of *gclc*, *gstp1*, *nqo-1*, *sod1*, and *sod2* increased significantly, as shown in Figure 1D. These results indicate that H_2_O_2_ and DEM activate downstream antioxidant target genes of the Nrf2/ARE signaling pathway by regulating the expression of *maft*, thus playing a protective role in zebrafish.

**Figure 2:**
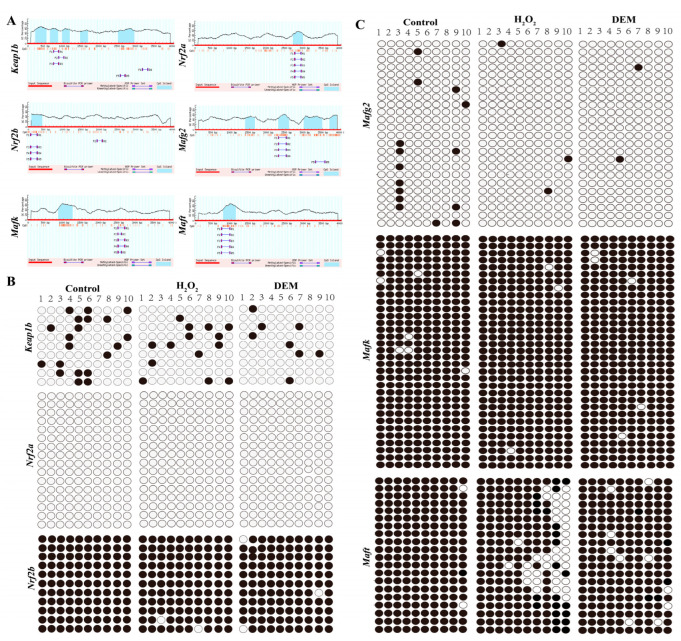
Methylation analysis of promoter regions of the Keap1 /Nrf2 signaling pathway-related genes before and after treatment. (**A**) Methylation prediction of promoter regions of genes related to the Keap1 /Nrf2 signaling pathway, Methylation analysis of promoter regions of (**B**) *keap1* and *nrf2*, (**C**) *Mafg2*, *Mafk*, and *Maft*. Each cycle is a CpG in the DNA sequence represented by either a closed circle (methylated cytosine) or an open circle (unmethylated cytosines). Each column of CpGs represents one colony sequenced, with a total of 10 positive clones.

By predicting CpG islands in the promoter regions of the Nrf2/ARE signaling pathway-related genes in zebrafish, it was found that the promoter regions of *keap1b*, *nrf2a*, *nrf2b*, *mafg2*, *mafk*, and *maft* contained CpG islands, as shown in Figure 2A. Genomic DNA was extracted from zebrafish larvae at the 120 hpf stage under different treatments. After treatment with a methylation kit, methylation primers were designed for methylation PCR, as shown in Figure S1. Changes in CpG island methylation were detected by sulfite sequencing in zebrafish under oxidative stress and normal conditions. Each cycle is a CpG in the DNA sequence, represented by a closed circle (methylated cytosine) or open circle (unmethylated cytosines). Each column of CpG represents one sequenced colony, with a total of 10 positive clones. The results showed that promoter CpG islands of *keap1b*, *nrf2a*, and *mafg2* were in a hypomethylation state regardless of the normal state or oxidative stress state, while *nrf2b* and *mafk* were in a hypermethylation state. This indicates that when zebrafish are stimulated by oxidant/electrophile, the upregulation of the expression of the five genes mentioned above is independent of the methylation in the promoter region, as shown in Figure 2B,C. Interestingly, the methylation of the promoter region of *maft* was significantly lower in the oxidative stress state than that in the normal state, as shown in Figure 2C, suggesting that changes in the expression of *maft* may be regulated by the methylation of its promoter region. The above results indicate that the activation of the Nrf2/ARE signaling pathway after being stimulated by oxidants and electrophiles is presumed to demethylate the *maft* promoter region, thereby regulating the expression of *maft*, activating downstream target gene transcription and protecting the body from external environmental damage.

### 3.2. H_2_O_2_ and DEM Activate Nrf2/ARE Signaling Pathway Mechanism in Zebrafish

In this experiment, six key points of development were selected, including 8 hpf, 12 hpf, 24 hpf, 48 hpf, 120 hpf, and 168 hpf. Taking the gene expression at 8 hpf as a reference, the expression of *maft* increased with the development of embryos, and the highest was at 120 hpf, as shown in Figure S2. In order to confirm that the demethylation of the *maft* promoter can regulate the expression of the *maft* and activate the transcription of the target genes, the demethylation of the *maft* promoter region of 120 hpf zebrafish was carried out. At present, azacitidine and decitabine were mainly used in the experimental studies of cells, but there were no relevant reports in zebrafish models.

**Figure 3:**
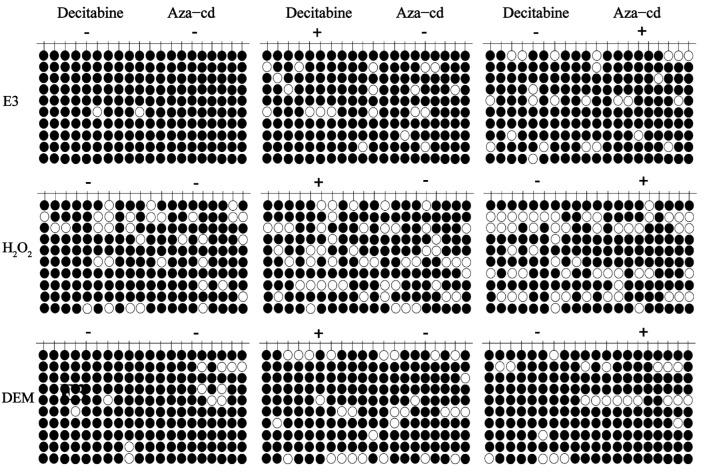
The level of methylation of *maft* promoter after inhibitors treatment. Each cycle is a CpG in the DNA sequence represented by either a closed circle (methylated cytosine) or an open circle (unmethylated cytosines). Each row of CpGs represents one colony sequenced, with a total of 10 positive clones.

Nine groups were designed, including E3 water control, 1 mmol/L H_2_O_2_, 100 µmol/L DEM (Diethyl Maleate), E3 water +3 µmol/L decitabine, 1 mmol/L H_2_O_2_ + 3 µmol/L decitabine, 100 µmol/L DEM + 3 µmol/L decitabine, E3 water +3 µmol/L azacitidine, 1 mmol/L H_2_O_2_ + 3 µmol/L azacitidine, and 100 µmol/L DEM + 3 µmol/L azacitidine for 6 h at 120 hpf. Next, the methylation of the CpG island in the *maft* promoter region was tested under different conditions. It was found that the rate of methylation in the E3 water group was 99%. The rate of methylation is 90% after E3 + decitabine treatment, and the rate of methylation is 87.5% after E3 + azacitidine treatment. Compared with the control group, decitabine and azacitidine significantly reduced the level of the methylation of the CpG island in the *maft* promoter region, indicating that both inhibitors effectively inhibited the *maft* promoter region methylation. The rate of methylation in the H_2_O_2_ group was 83.5%, the rate of methylation in the H_2_O_2_ + decitabine treatment was 74.5%, and the rate of methylation in the H_2_O_2_ + azacitidine treatment was 72.5%. Compared with the control group without inhibitors, the degree of the *maft* promoter region methylation was reduced after decitabine and azacitidine treatment, indicating that H_2_O_2_ had a synergistic effect with the two inhibitors in demethylation. The rate of methylation in the DEM group was 94%, the methylation rate was 85.5% after DEM + decitabine treatment, and the methylation rate after DEM + azacitidine treatment was 89%. Compared with the control group without inhibitors, the *maft* promoter region methylation was reduced after decitabine and azacitidine treatment, indicating that DEM and the two inhibitors had a synergistic effect on demethylation. The demethylation effect was shown in Figure 3. Each cycle is a CpG in the DNA sequence represented by either a closed circle (methylated cytosine) or an open circle (unmethylated cytosines). Each row of CpG represents one colony sequenced, with a total of 10 positive clones. The above results suggest that H_2_O_2_ and DEM cooperate with decitabine and azacitidine to promote the demethylation of the *maft* promoter.

**Figure 4:**
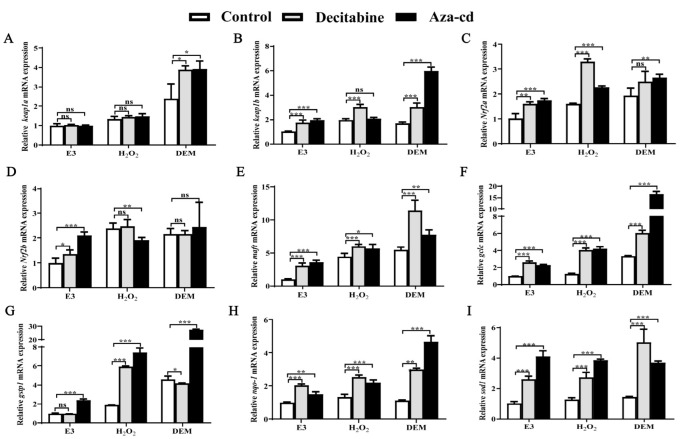
The expressions of the Nrf2/ARE signaling pathway-related genes and target genes under different stimuli after the addition of decitabine and azacytidine (**A**) *keap1a*, (**B**) *keap1b*, (**C**) *nrf2a*, (**D**) *nrf2b*, (**E**) *maft*, (**F**) *gclc*, (**G**) *gstp1*, (**H**) *nqo-1*, (**I**) *sod1*. n = 30, *** *p* < 0.001, ** *p* < 0.01, * *p* < 0.05, ns *p* > 0.05, mean ± SEM.

To verify whether Nrf2/ARE signaling activation was regulated by *maft* promoter demethylation, we used qRT-PCR methods to detect the expression of Nrf2/ARE signaling-related genes and their target genes. We found that the expression of *keap1a*, *keap1b*, *Nrf2a*, and *Nrf2b* was upregulated after the addition of decitabine and azacitidine compared to the E3 control group, as shown in Figure 4A–D. We found the expression of *maft* was upregulated after the demethylation of the *maft* promoter by decitabine and azacitidine. It activated the transcription of the downstream antioxidant genes *gclc*, *gstp1*, *nqo-1*, and *sod1*, as shown in Figure 4E–I. The above results indicate that decitabine and azacitidine promote the *maft* promoter demethylation, thereby promoting *maft* expression, and activating the transcription of the antioxidant target genes of the Nrf2/ARE signaling pathway.

**Figure 5:**
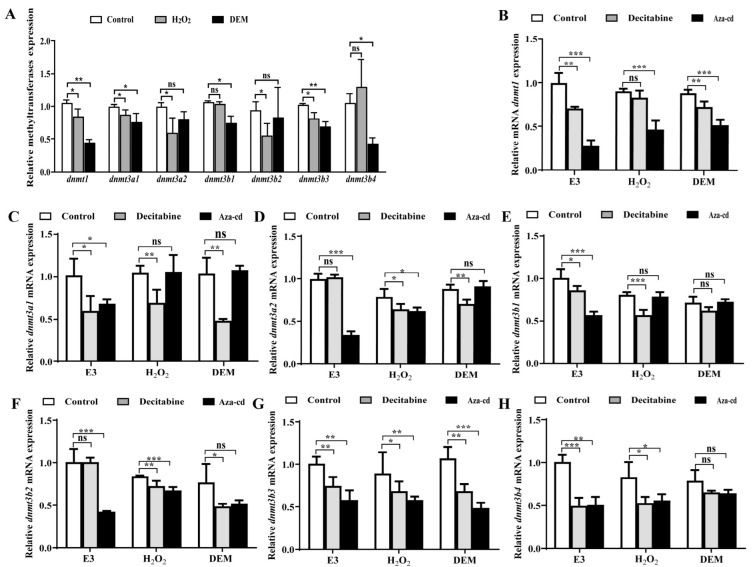
Analysis of methyltransferase expression (**A**) after H_2_O_2_/DEM treatment, after methyltransferase inhibitors treatment, analysis of the expression of (**B**) *dnmt1*, (**C**) *dnmt3a1*, (**D**) *dnmt3a2*, (**E**) *dnmt3b1*, (**F**) *dnmt3b2*, (**G**) *dnmt3b3*, (**H**) *dnmt3b4*. n = 30, *** *p* < 0.001, ** *p* < 0.01, * *p* < 0.05, ns *p* > 0.05, mean ± SEM.

In order to explore the regulatory mechanism of methylation changes in CpG island in the maft promoter, the expression of seven DNA methyltransferases from the above nine treatment groups was detected at 120 hpf in zebrafish. Under H_2_O_2_ stimulation, the expression of *dnmt1*, *dnmt3a1*, *dnmt3a2*, *dnmt3b2*, and *dnmt3b3* decreased, while the expression of *dnmt3b1* and *dnmt3b4* did not change significantly. The expression of the seven methyltransferases was all downregulated under DEM stimulation, as shown in Figure 5A. When treated with decitabine, the expression of methyltransferases except dnmt1 was all decreased under the stimulation of H_2_O_2_. When treated with azacitidine, only the expression of *dnmt1* and *dnmt3b3* was downregulated under DEM stimulation, as shown in Figure 5B–H. The overall expression of methyltransferase decreased under the inhibitor treatment, even though different inhibitor treatments showed different results. These results suggest that H_2_O_2_/DEM have similar functions with decitabine and azacitidine, both of which promote the demethylation of the promoter of *maft* by inhibiting methyltransferase expression, and increase the expression of *maft*, thereby activating the transcription of downstream antioxidant genes of the Nrf2/ARE signaling pathway.

## 4. Discussion

The antioxidant response, anti-inflammatory, and cellular protective properties of the Nrf2/ARE signaling pathway are some of the major means of cellular defense. Although many diseases are closely related to the disorder of the Nrf2/ARE signaling pathway, studies on the whole Nrf2/ARE signaling pathway-related genes under early environmental stimulation are rarely reported in zebrafish [18,19,20,21]. H_2_O_2_/DEM stimulated early zebrafish embryos, and it is found that the Nrf2/ARE signaling pathway is activated (Figure 1). The expression of Keap1a and Keap2 are found to increase during this process. At present, we cannot explain the reason for this phenomenon, which needs further study. H_2_O_2_/DEM stimulated the generation of reactive oxygen species, such as hydroxyl radicals, and can cause serious DNA damage and mutation, which can also lead to DNA hypomethylation [17]. DNA methylation of the Nrf2/ARE signaling pathway-related genes has been deeply studied in humans and mice, but this mechanism needs to be further studied in zebrafish. Taking the gene expression at 8 hpf as a reference, the expression of *maft* increased with the development of embryos, and the highest was at 120 hpf. It was consistent with the time expression profile of *maft* in developing embryos found in previous research [22]. At the same time, it was consistent with the zebrafish period selected in the previous study. In order to confirm that the demethylation of the *maft* promoter can regulate the expression of the *maft* and activate the transcription of the target genes, the demethylation of the *maft* promoter region of 120 hpf zebrafish was carried out. We demonstrated that H_2_O_2_/DEM demethylates the Maft promoter region (Figure 2). Azacitidine is a cytosine analogue that has the unique ability to covalently capture DNA methyltransferase, depriving it of its activity to modify cytosine to 5-methyl cytosine [23]. Decitabine is a deoxyribonucleotide that is directly phosphorylated and incorporated into DNA to inhibit DNA methylation [24]. Both are demethylated by the incorporation of DNA and the covalent capture of DNA methyltransferase. DNA methyltransferase, as the carrier of methyl on the fifth carbon atom of the deoxycytosine ring, plays an important role in DNA methylation. The expression of methyltransferase decreased under the inhibitor treatment. However, we had not explored the mechanism. This study only suggested that H_2_O_2_/DEM had similar functions with decitabine and azacitidine, both of which promoted the demethylation of the promoter of *maft* by inhibiting methyltransferase expression. Meanwhile, it increased the expression of *maft*, thereby activating the transcription of the downstream antioxidant genes of the Nrf2/ARE signaling pathway, as shown in Figure 4 and Figure 5.

## 5. Conclusions

We predicted the CpG islands in the promoter region of genes related to the zebrafish Nrf2/ARE signaling pathway and performed methylation analysis. First, zebrafish larvae were exposed to H_2_O_2_ and DEM, and the expression of the Nrf2/ARE signaling pathway-related genes was found to be significantly upregulated. Subsequently, CpG islands in the promoter regions of *keap1b*, *nrf2a*, and *mafg2* were found to be hypomethylated under both normal and oxidative stress states, while *nrf2b* and *mafk* were hypermethylated. These results indicate that the upregulation of the expression of these five genes is not related to promoter methylation when the zebrafish larvae are exposed to oxidants/electrophiles. Interestingly, the methylation of the *maft* promoter region was significantly reduced in the oxidative stress state than in the normal state. It suggested that changes in the expression of *maft* were regulated by methylation in its promoter region. Next, methyltransferase inhibitors (decitabine, azacitidine) were used to demethylate the promoter region of the *maft* in normal and oxidative stress states. The results showed that H_2_O_2_ and DEM had synergistic effects with methyltransferase inhibitors, and the methylation degree of the *maft* promoter was further reduced. Our studies have shown that the inhibition of methyltransferase expression can lead to the demethylation of the maft promoter, thereby promoting the expression of *maft*, activating the expression of Nrf2/ARE signaling pathway-related genes and downstream target genes, and ultimately maintaining homeostasis of the internal environment. Our new findings can target the methylation of the *maft* promoter to treat a variety of antioxidant signaling-related diseases and will provide new strategies for the treatment of cancer, aging, cardiovascular disease, and neurodegenerative diseases.

## Data Availability

All data generated or analyzed during this study are included in this published article.

## Supplementary Materials

The following supporting information can be downloaded at: https://www.mdpi.com/article/10.3390/life12091436/s1, Figure S1. Gel electrophoresis of methylated PCR products; Figure S2. The expression of *maft* in different periods; Table S1. PCR Oligonucleotide primers; Table S2. Methylated PCR primers.
