# Supplementary material for: H2O2/DEM-Promoted Maft Promoter Demethylation Drives Nrf2/ARE Activation in Zebrafish
Source: Life (Basel). 2022 Sep 15;12(9):1436. doi: 10.3390/life12091436 (PMC9503143; doi:10.3390/life12091436)
Supplement: Supplementary file 1 [file life-12-01436-s001.zip › life-1862767-supplementary.pdf]

Article

H_2O_2/DEM-promoted *maft* promoter demethylation drives Nrf2/ARE activation in zebrafish

Ce Chen[1#], Mingyue He[1#], Xueting Li[1#], Lidong Yu[2], Yi Liu[1], Yan Yang[1], Li Li[1*], Jianbo Jia[3*], Bingsheng Li[4]

1 School of Life Science and Technology, Harbin Institute of Technology, Harbin 150080, P.R. China
2 School of Physics, Harbin Institute of Technology, Harbin 150080, P.R. China
3 School of Biotechnology and Health Sciences, Wuyi University, Jiangmen 529020, P.R. China
4 Key Laboratory of UV Light Emitting Materials and Technology of Ministry of Education, Northeast Normal University, Changchun 130024, P. R. China
# These authors contributed equally.
* Correspondence: lilili@hit.edu.cn (L.L.); jbjia@wyu.edu.cn (J.J.)

Table S1. PCR Oligonucleotide primers.

Genes	Forward Primer sequences	Reverse Primer sequences
keap1a	5'- GTGTGGAGTGCTACTGTCCC -3'	5'- TCCTCCTCTGGCAGGATACC -3'
Keap1b	5'- ATCGAGGGGATACACCCCAA -3'	5'- AGCTGCTCCACCAGGAAATC -3'
nrf2a	5'- GAGCGGGAGAAATCACACAGAATG -3'	5'- CAGGAGCTGCATGCACTCATCG -3'
nrf2b	5'- GCCACGTTATGCTGGGTTTC -3'	5'- CTGCGGACAACGATAGCAGA -3'
mafk	5'- ACCTGTTGACTCCAGGACACT -3'	5'- AAATCTGTCTTGTGAACGCGG -3'
mafg1	5'- TCATTATAAGGGCGCTGTGGG -3'	5'- GACTCGTGCCATTCTCACCT -3'
mafg2	5'- CGCTCCAAATACGAGGCTCT -3'	5'- GGCGTCGGTCTTGGACTTTA -3'
maft	5'- GTGTCAGACGAAACACCGGA -3'	5'- CCCTTTTCACCTTGAGGGCT -3'
gclc	5'- AACCGACACCCAAAGATTCAGCACT -3'	5'- CCATCATCCTCTGGAAACACCTCC -3'
gstp1	5'- CGACTTGAAAGCCACCTGTGTC -3'	5'- CTGTCGTTTTTGCCATATGCAGC -3'
ef1α	5'- CCTGGGAGTGAAACGCTGATC -3'	5'- CCGATCTTCTTGATGTATGCGCTG -3'
nqo1	5'- TTTGCAGAATCCCGAGCACT -3'	5'- TCTTCTGCGATCAAGCTGAAAG -3'
sod1	5'- CTAGCCCGCTGACATTACATC -3'	5'- TTGCCCACATAGAAATGCAC -3'
sod2	5'- CGCATGTTCCCAGACATCTA -3'	5'- GAGCGGAAGATTGAGGATTG -3'
dnmt1	5'-GGGCTACCAGTGCACCTTTG -3'	5'- GATGATAGCTCTGCGTCGAGTC -3'
dnmt3a1	5'- TTTTCGGTTTGTTTTTTGTTC -3'	5'- CCGTCACCGATCAATAACT -3'
dnmt3a2	5'- ATGTGAATATTGATGTTTTGGTAGTGT -3'	5'- AATAAACAACCACATTCCAAATTAAA -3'
dnmt3b1	5'- AGTGTGTATGATATTATTTATTTGTTTGTG -3'	5'- ACATTAATCTTTCTTCCTCTTCAAATC -3'
dnmt3b2	5'- GTGGATTTTTTATGAATGAAATTA -3'	5'- TACACACTCAACAAAACACAAAAAC -3'
dnmt3b3	5'- AGGTGAGAATGTTTCGGC -3'	5'- AACGACCCACGAATAACAAT -3'
dnmt3b4	5'- TTTTTGTTATAAAATATTTTTTTTGTT -3'	5'- CCTTCTTCTACTACTCCAAAACCTC -3'

Table S2. Methylated PCR primers

Genes	Forward Primer sequences	Reverse Primer sequences
keap1a	5'- TTTTCGGTTTGTTTTTTGTTC -3'	5'- CCGTCACCGATCAATAACT -3'
nrf2a	5'- ATGTGAATATTGATGTTTTGGTAGTGT -3'	5'- AATAAACAACCACATTCCAAATTAAA -3'
nrf2b	5'- AGTGTGTATGATATTATTTATTTGTTTGTG -3'	5'- ACATTAATCTTTCTTCCTCTTCAAATC -3'
mafg2	5'- GTGGATTTTTTATGAATGAAATTA -3'	5'- TACACACTCAACAAAACACAAAAAC -3'
mafk	5'- AGGTGAGAATGTTTCGGC -3'	5'- AACGACCCACGAATAACAAT -3'
maft	5'- TTTTTGTTATAAAATATTTTTTTTGTT -3'	5'- CCTTCTTCTACTACTCCAAAACCTC -3'

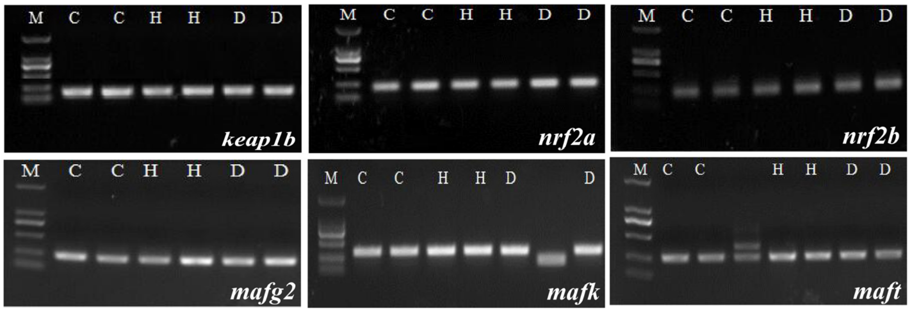

Figure S1. Gel electrophoresis of methylated PCR products.

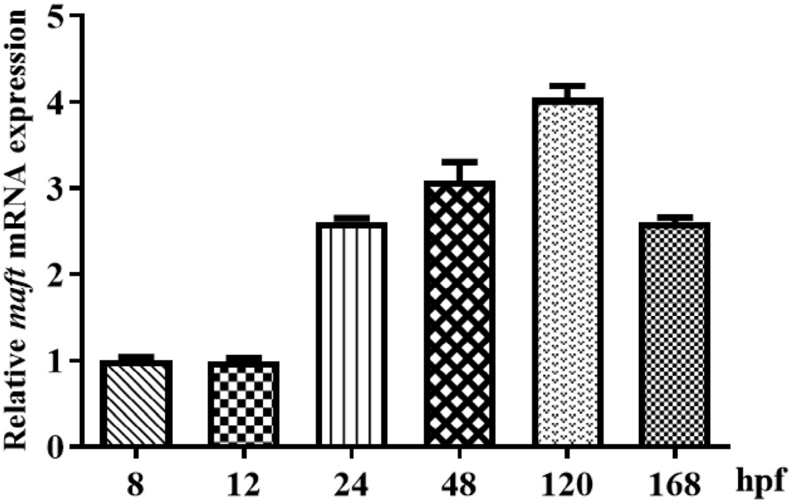

Figure S2. The expression of maft in different periods.
